# Analysis of clinical features and outcomes in a Chinese cohort with *ALK* fusion across pan-cancer: a retrospective study

**DOI:** 10.3389/fonc.2026.1890263

**Published:** 2026-09-03

**Authors:** Meng Zhang, Yi Feng, Xue Du, Changda Qu, Meizhu Meng, Meiying Ye, Min Liang, Ziran Yang, Wenjuan Gong, Xingyu Ma, Jialiang Guo, Wenmei Li, Shuqin Jia

**Affiliations:** 1 Key laboratory of Carcinogenesis and Translational Research (Ministry of Education/Beijing), Center for Molecular Diagnostics, Peking University Cancer Hospital & Institute, Beijing, China; 2 State Key Laboratory of Holistic Integrative Management of Gastrointestinal Cancers, Beijing Key Laboratory of Carcinogenesis and Translational Research, Center for Molecular Diagnostics, Peking University Cancer Hospital & Institute, Beijing, China

**Keywords:** anaplastic lymphoma kinase (ALK), chromosomal fusion, concomitant mutations, fusion, treatment outcome, tyrosine kinase inhibitor (TKI)

## Abstract

**Background:**

*ALK* fusions are validated oncogenic drivers, yet their molecular heterogeneity and therapeutic implications remain poorly characterized. This study defined the landscape of *ALK* fusion partners, *EML4-ALK* variants, and co-mutations and their impact on treatment responses in a large Chinese pan-cancer cohort.

**Methods:**

We retrospectively analyzed 16,335 consecutive pan-cancer patients via next-generation sequencing to identify *ALK* fusions. Clinical characteristics, fusion partners, co-mutations, and treatment responses were evaluated. Progression-free survival (PFS) was assessed in advanced non-small cell lung cancer (NSCLC) patients receiving first-line *ALK* tyrosine kinase inhibitors (TKIs), stratified by *EML4-ALK* variants and *TP53* status.

**Results:**

*ALK* fusions were detected in 321 patients. NSCLC accounted for 93.1% (299/321), predominantly adenocarcinomas (292/299, 97.7%). *EML4* was the predominant fusion partner, accounting for 92.1% of *ALK* fusion in lung adenocarcinoma but only 27.3% in extrapulmonary tumors. Conversely, non-*EML4* fusions were more frequent in extrapulmonary tumors (72.7%). Among 13 evaluable advanced-stage patients with rare *ALK* fusions, 10 achieved objective response (76.9%). In 161 advanced or recurrent NSCLC patients treated with *ALK*-TKIs, the median PFS was 28.2 months. Patients with long *EML4-ALK* variants had superior PFS than those with short variants when treated with second-generation TKIs (55.7 vs. 27.1 months; *p* = 0.019). *TP53* co-mutations were associated with shorter PFS in the first-generation TKI subgroup (5.6 vs. 21.4 months; *p* = 0.009).

**Conclusions:**

While *ALK* fusion partners exhibit tumor-type specificity, *ALK*-TKIs demonstrate potential efficacy in rare fusions though these findings are exploratory and need validation. Short *EML4-ALK* variants and *TP53* co-mutation are associated with poorer TKI efficacy.

## Introduction

Anaplastic lymphoma kinase (*ALK*) gene alterations, including activating mutations, amplifications, and fusion, have become critical biomarkers across diverse malignancies (1). Although *ALK* fusions occur in 3% - 7% of non-small cell lung cancer (NSCLC), their prevalence varies markedly between tumor types, ranging from greater than 80% in anaplastic large cell lymphoma to less than 1% in breast and colorectal cancers (1). This molecular heterogeneity extends to fusion partner diversity, with more than 90 distinct partners identified to date (2). In NSCLC, the *EML4* (echinoderm microtubule-associated protein-like 4)*-ALK* fusion accounts for approximately 85% of *ALK*-positive patients (3), whereas *EML4-ALK* fusions are found in only about 31% of *ALK*-positive extrapulmonary malignant tumors (1). Crucially, the breakpoint location of *EML4* influences both functional consequences and therapeutic responsiveness. The most common variants are *EML4-ALK* variant 1 (E13:A20) and variant 3a/b (E6:A20), comprising 43% and 40% of occurrences, respectively (4). Emerging evidence suggests that variant 3a/b correlate with adverse prognosis (5–8). Concurrent *TP53* mutations and resistance mutations within the *ALK* gene are also implicated in accelerated resistance to *ALK* tyrosine kinase inhibitors (TKIs) (5, 8–10).

The number of therapeutic strategies against *ALK*-driven malignancies has expanded rapidly, with three generations of TKI now available. First-generation inhibitor crizotinib has demonstrated proof-of-concept efficacy but faces limitations in blood-brain barrier penetration and resistance mutation coverage. Second-generation agents, including ceritinib, alectinib, brigatinib, ensartinib, and iruplinalkib improve central nervous system activity. The third generation inhibitor lorlatinib exhibits broader resistance mutation coverage and superior intracranial disease control (11, 12). Nevertheless, differential responses across fusion variants, and the prognostic impact of concurrent mutations remain incompletely characterized. Furthermore, current evidence derives predominantly from NSCLC cohorts, leaving critical gaps in understanding *ALK* biology across rarer malignancies.

In this retrospective study, we screened out 321 patients with *ALK* fusions from 16,335 pan-cancer patients via next generation sequencing (NGS), and systematically evaluated the genomic landscape of *ALK* fusions beyond NSCLC, variant-specific outcomes stratified by TKI generations, and the synergistic impact of co-mutations on therapeutic resistance. By integrating molecular profiling with longitudinal clinical data, this work aims to refine predictive models for *ALK*-targeted therapies and inform personalized treatment decisions.

## Materials and methods

### Study design and cohort characteristics

**Figure 1:**
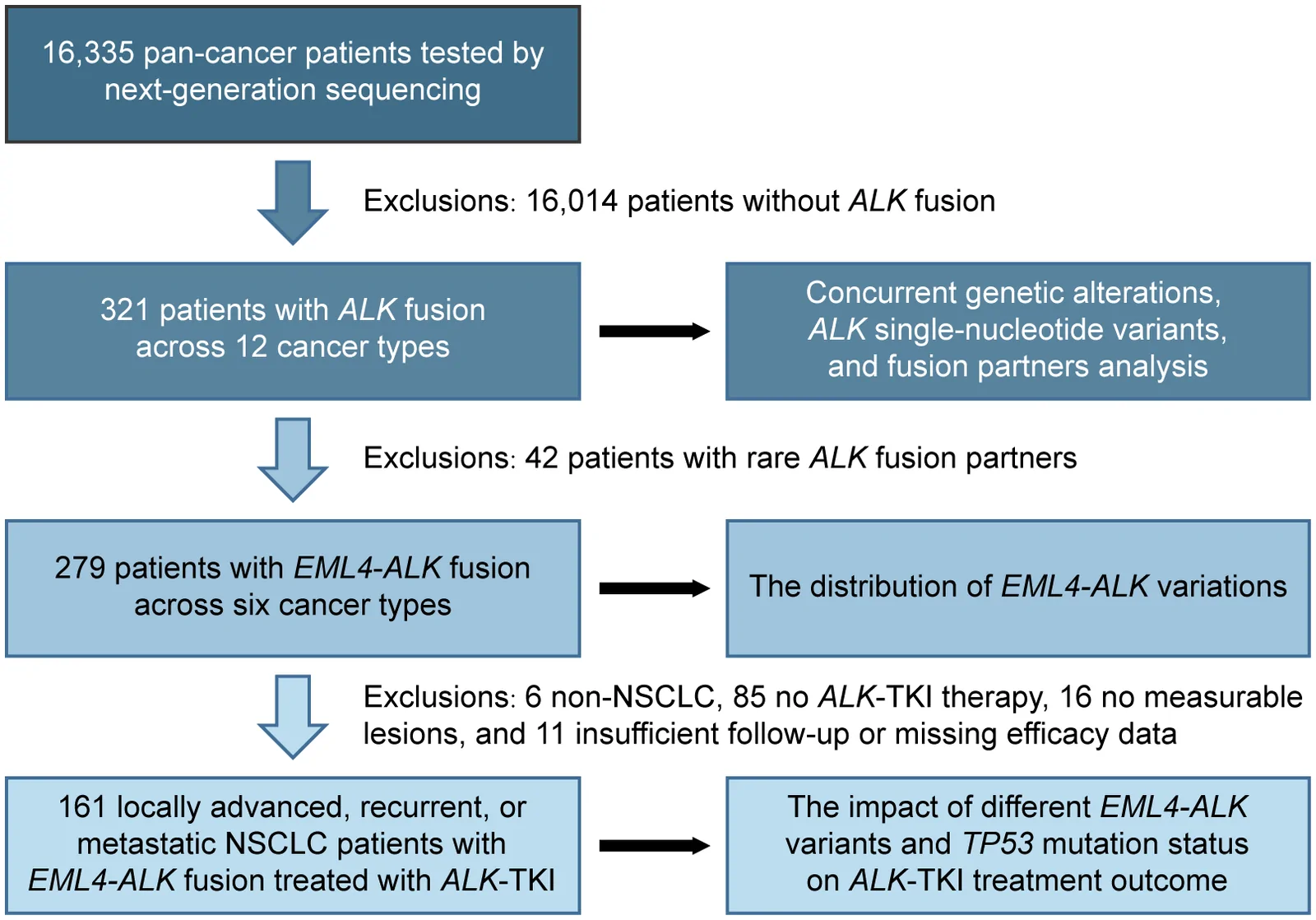
Study flowchart with patient enrollment, exclusion steps, and final analytic cohort.

This retrospective cohort study analyzed NGS data from 16,335 consecutive patients with histologically confirmed malignancies treated at Peking University Cancer Hospital & Institute between April 2016 and April 2024 (**Figure 1**). Inclusion criteria required: age 18 years or older at diagnosis, confirmation of *ALK* fusion positivity through NGS at either DNA or RNA level, and availability of clinical records including treatment response assessment. Patients with ambiguous histopathological diagnoses were excluded from the analysis. Clinical characteristics, including age, sex, smoking status, histological subtype, clinical stage according to the American Joint Committee on Cancer (AJCC) 8th edition, treatment regimens, and metastatic sites were systematically extracted from electronic health records. Written informed consent was obtained from all participants before the NGS test, and the study was approved by the Medical Ethics Committee of the Peking University Cancer Hospital & Institute (2016XJS01). Progression-free survival (PFS) was calculated as the time from the initiation of first-line *ALK*-TKI therapy to radiological or clinical progression according to the Response Evaluation Criteria in Solid Tumors (RECIST) version 1.1 or to all-cause mortality. Progression events were identified from routine clinical imaging reports and medical records documented by treating physicians. Patients were censored at the date of the last evaluable tumor assessment if they had not progressed; at the date of last follow-up for those lost to follow-up; at the start of subsequent systemic therapy; or at the last evaluable assessment before missed visits for those with incomplete radiological evaluations.

### Statistical analysis

Categorical variables were compared via the *χ*² test or Fisher’s exact test. Continuous variables were reported as medians with interquartile ranges (IQRs) and analyzed with the Mann-Whitney *U* test. PFS was displayed using Kaplan-Meier curves generated by the SurvMiner R package, and comparisons were made using the log-rank test. For the multiple pairwise log-rank tests, we applied the Holm-Bonferroni correction to control the family-wise error rate. Both unadjusted and adjusted *p* values were reported, with statistical significance determined by adjusted *p* value. Multivariate Cox proportional hazards models were used to derive hazard ratios (HRs). All analyses were performed using R 4.3.2, and SPSS version 22, with statistical significance defined as two-sided *p* < 0.05.

## Results

### Demographics and clinical characteristics

*ALK* fusions were identified in 321 (1.97%) of 16,335 consecutive tumor specimens (**Figure 1**). The cohort demonstrated a female predominance (193 females vs. 128 males; female-to-male ratio 1.51:1), with a median age of 53 years (range: 20 – 86). NSCLC accounted for 93.1% of cases (299/321), predominantly comprising adenocarcinomas (292/299, 97.7%). Other NSCLC pathological types included three cases of lung squamous cell carcinoma (LUSC) and four cases of other histologies (adenosquamous, mucoepidermoid, large cell, and carcinoid tumor). Extrapulmonary malignancies (22/321, 6.9%) spanned 11 distinct cancer types, with melanoma (n = 6), sarcoma (n = 5), and colorectal carcinoma (n = 3) being most frequent; the remaining eight malignancies each occurred as single cases (**Supplementary Table 1**).

### Co-mutation landscape and *ALK* fusion partners distribution

**Figure 2:**
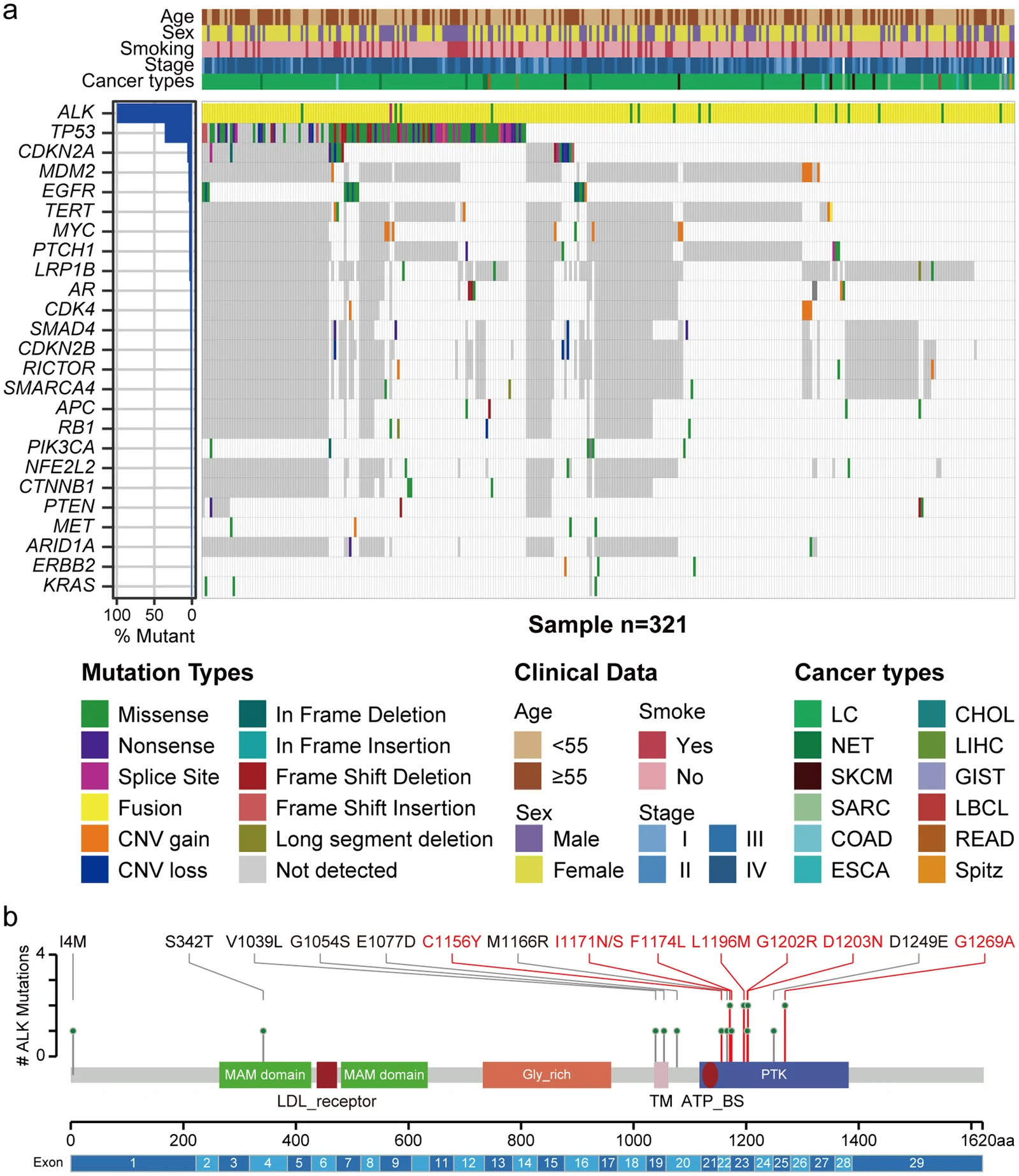
Summary of patient characteristics, mutation landscape and the SNVs localization in *ALK*. **(a)** An integrated overview of patient demographics and genomic alterations in the *ALK* fusion-positive cohort. Patients were classified by age range, sex, smoking history, clinical stage, and cancer type. Due to the different panels used for testing, the frequency of co-mutated genes was calculated on the basis of patients with available sequencing data for each gene. **(b)** Schematic diagram of the *ALK* gene domains and the number and localization of SNVs. The schematic highlights known ALK tyrosine kinase inhibitor resistance mutations, which are distinctly marked in red based on their previous documentation in the published literature. The lower part of the figure represents the exons encoding the *ALK* gene. CNV, copy number variation; LC, lung cancer; CHOL, cholangiocarcinoma; NET, neuroendocrine tumor; LIHC, liver hepatocellular carcinoma; SKCM, skin cutaneous melanoma; GIST, gastrointestinal stromal tumor; SARC, sarcoma; LBCL, large B-cell lymphoma; COAD, colon adenocarcinoma; READ, rectum adenocarcinoma; ESCA, esophageal carcinoma; Spitz, atypical Spitz tumor; MAM, meprin, A5 protein and protein tyrosine phosphatase Mu domain; LDL, low-density lipoprotein receptor domain; Gly-rich, glycine-rich region; TM, transmembrane helix; ATP_BS, adenosine triphosphate binding site; PTK, protein tyrosine kinase domain.

Owing to the different panels used for NGS testing, the frequencies of co-mutated genes were calculated on the basis of patients with available sequencing data for each gene, and the gene-specific denominators were provided in **Supplementary Table 2**. *TP53* mutations were the most prevalent concurrent genomic alteration (36.2%, 101/279), followed by *CDKN2A* (6.1%, 16/262), *MDM2* (4.7%, 6/129), and *EGFR* (4.4%, 14/321, **Figure 2a**). Notably, 5.0% of patients (16/321) harbored concurrent *ALK* single-nucleotide variants (SNVs) with three patients harboring two distinct *ALK* mutations simultaneously. Molecular mapping revealed seven specific resistance-conferring mutations within the *ALK* protein kinase domain: C1156Y, I1171N/S, F1174L, L1196M, G1202R, D1203N, and G1269A (**Figure 2b**). Nine patients carried the above-mentioned *ALK*-resistant mutations.

**Figure 3:**
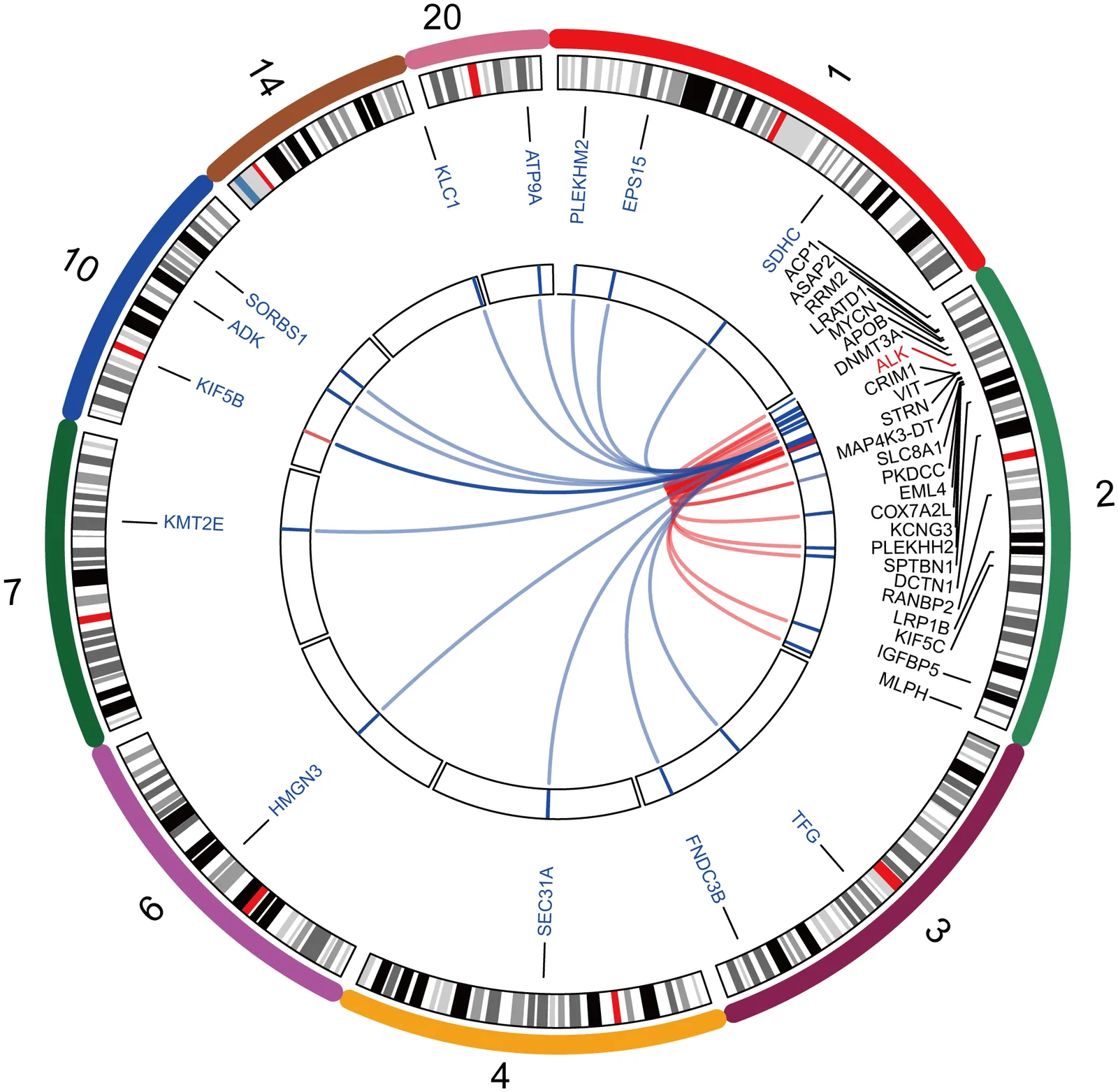
Distribution of *ALK* gene fusion partners and chromosomal location. This circular visualization depicts the distribution patterns of *ALK* fusion partners throughout the human genome. The plot features a multi-tiered design with the *ALK* gene (highlighted in red) locus at chromosome 2p23.2. Intra-chromosomal fusion partners (located on chromosome 2) are labeled in black text and connected to *ALK* via red curved links in the innermost layer, while inter-chromosomal partners are displayed in blue text with corresponding blue connecting arcs. The middle ring features a gradient heatmap that visualizes fusion event frequencies, revealing a striking predominance of *EML4-ALK* fusions (n=279) over other partners. While *KIF5B* (n=5) and *DCTN1* (n=2) demonstrate recurrent but less frequent involvement, the remaining partners each occurred as single events. The outermost ring provides genomic context by mapping each partner gene to its chromosomal location.

*EML4* emerged as the predominant *ALK* fusion partner (279/321, 86.9%), with 269 cases occurring in lung adenocarcinoma (LUAD) (**Supplementary Table 3**). Non-*EML4* fusions involved 37 distinct molecular partners, including recurrent *KIF5B*-*ALK* (n = 5) and *DCTN1*-*ALK* (n = 2), alongside 35 partners each identified once (**Figure 3**; **Supplementary Table 1**). The fusion partners on chromosome 2 were most common in terms of both quantity (303/321) and variety (24/38). Strikingly, non-*EML4* fusions were significantly more common in extrapulmonary malignancies than in LUAD patients (72.7% vs. 7.9%; *p* < 0.001, **Supplementary Table 3**). Demographic analyses revealed no significant differences in sex distribution (*p* = 0.932), median age (*p* = 0.407), or smoking status (*p* = 0.977) between the fusion subtypes (**Supplementary Table 3**).

### The distribution of *EML4*-*ALK* fusion variants

**Figure 4:**
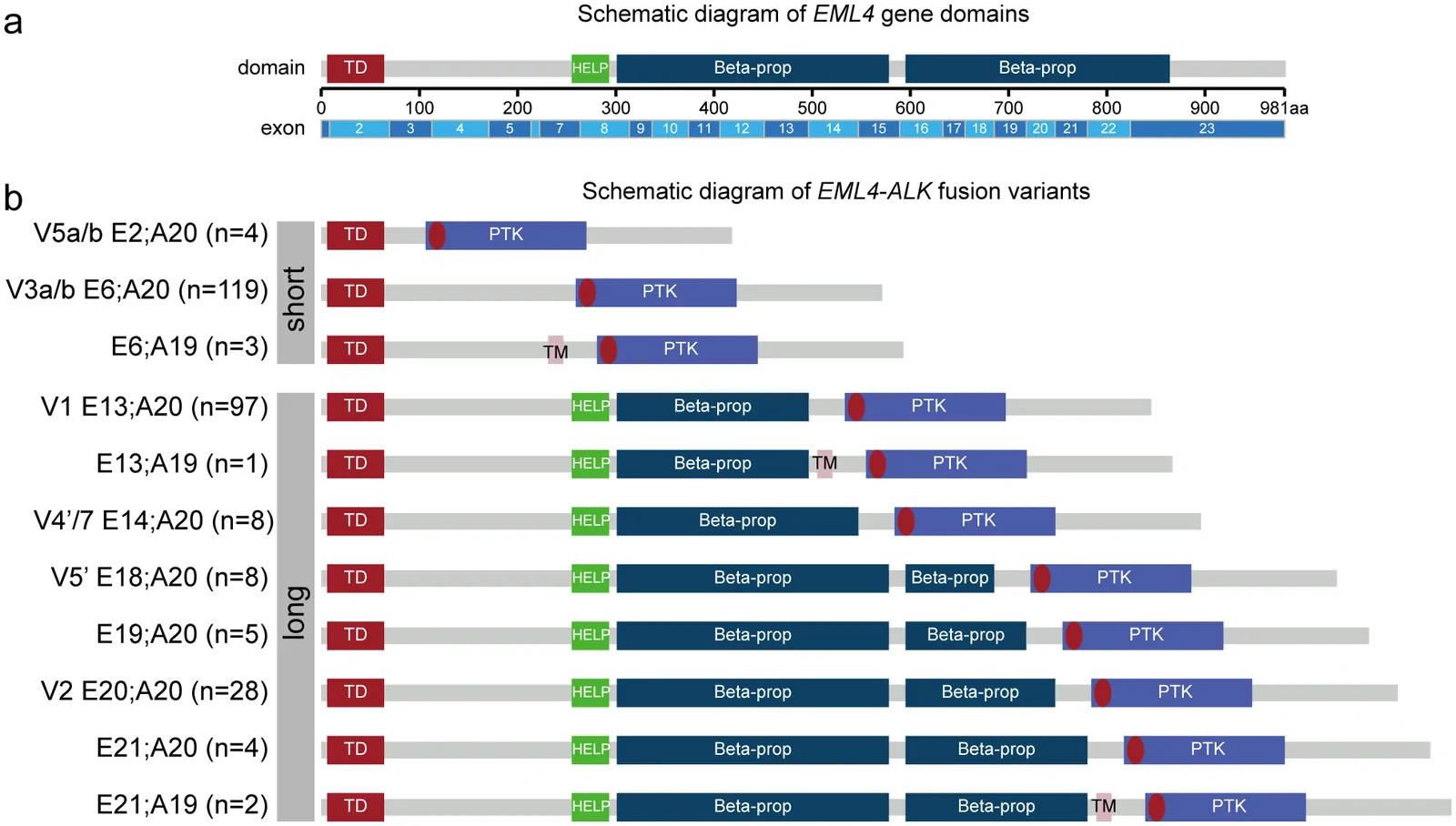
Schematic diagram of *EML4* gene domains and *EML4-ALK* fusion variants. **(a)** The schematic illustrates the domain architecture of the full-length *EML4* gene, with exons represented along the lower portion of the panel. Key functional domains are annotated, including the N-terminal trimerization domain (TD), the central hydrophobic EML protein motif (HELP), and the C-terminal β-propeller domain (β-prop), which mediate protein-protein interactions. **(b)** The panel depicts eleven characterized *EML4*-*ALK* fusion variants, demonstrating the variable breakpoints at which the *ALK* tyrosine kinase domain is fused to truncated *EML4* segments. Each variant retains the *ALK* kinase domain while incorporating different portions of the *EML4* N-terminus, which influences protein oligomerization and oncogenic potential. Long variants retaining the HELP and β-propeller domains. The red ovals in the PKT domain represent the ATP-binding site. TD, trimerisation domain; HELP, hydrophobic motif found in EML proteins; β-prop, β-propeller domain; TM, transmembrane helix; PTK, protein tyrosine kinase domain.

Eleven *EML4-ALK* fusion variants were identified in this study. Among them, three variants (45.2% of the total isoforms) lacked both the HELP and β-propeller domains of *EML4* (**Figure 4a**), producing truncated fusion proteins (**Figure 4b**). Among these short-form variants, the variant 3a/b isoform dominated, accounting for 94.4% (119/126) of the cases. The remaining eight isoforms retained the HELP domain essential for microtubule interaction and the β-propeller domain implicated in protein oligomerization. Within this long-form group, the canonical variant 1 (E13:A20) emerged as the predominant subtype, representing 63.4% (97/153) of the cases. Interestingly, patients with long variants had a three-years earlier age of onset compared to those with short variants (*p* = 0.006, **Supplementary Table 4**).

### Treatment outcomes in patients with rare *ALK* fusion partners

Among the 42 patients with non-*EML4* fusions, 16 patients received *ALK*-TKIs therapy. Of these, 3 patients received postoperative adjuvant therapy and were excluded from objective response rate (ORR) calculation due to the absence of measurable lesions. The remaining 13 patients had advanced or metastatic disease and received *ALK*-TKIs as their systemic treatment, including crizotinib, alectinib, ensartinib, brigatinib, or lorlatinib. Given the small and heterogeneous nature of this cohort, the ORR was calculated based on the best overall response to any *ALK*-TKI during the entire treatment course, regardless of treatment line, in order to provide a descriptive summary of observed clinical activity. Among the 13 evaluable patients, 10 achieved a partial response (PR), yielding an ORR of 76.9% (10/13). The duration of response ranged from 1.1 to over 55.8 months (**Supplementary Table 5**).

### Impact of *EML4-ALK* fusion variants and *TP53* mutations on PFS in patients with *ALK* fusion-positive NSCLC

**Figure 5:**
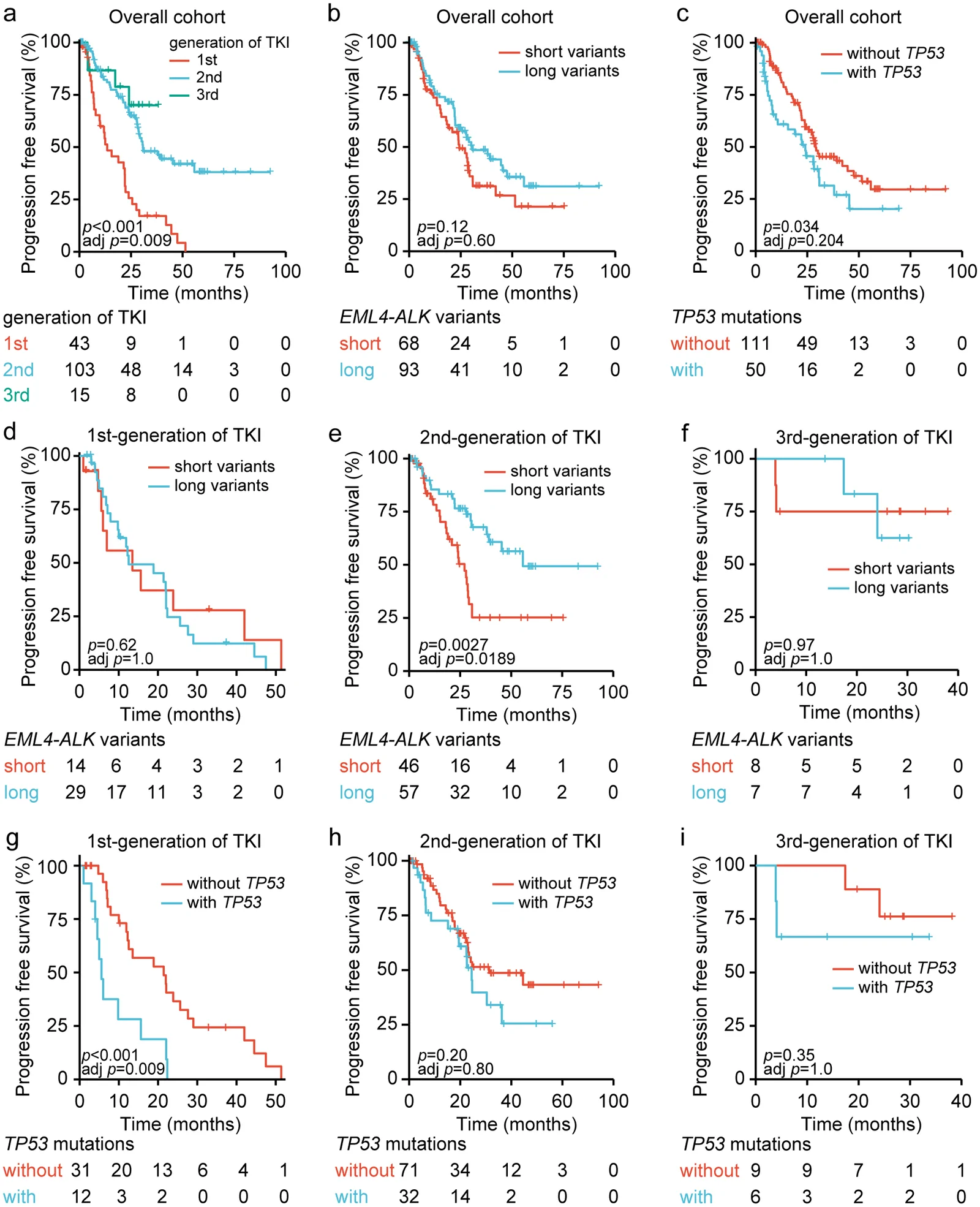
Impact of the molecular characteristics and generation of *ALK*-TKIs on the clinical outcomes of patients with *EML4-ALK* NSCLC. Figures present Kaplan-Meier analyses of progression-free survival (PFS) in patients with locally advanced, recurrent, or metastatic *EML4*-*ALK* fusion NSCLC. PFS comparisons across TKI generations **(a)**, *EML4*-*ALK* fusion variants **(b)**, and *TP53* mutation statuses **(c)**. Panels **(d–f)** show PFS stratified by different *EML4*-*ALK* variants within first-, second-, and third-generation TKI subgroups, respectively. Panels **(g–i)** show PFS stratified by *TP53* mutation status within first-, second-, and third-generation TKI subgroups, respectively. Both unadjusted log-rank *p* values and Holm-Bonferroni-adjusted *p* values are shown. Statistical significance was determined by adjusted *p* < 0.05. Subgroups with small sample sizes such as third-generation TKI groups (n=15) are presented descriptively without formal statistical inference. PFS, progression-free survival; NSCLC, non-small cell lung cancer; TKI, tyrosine kinase inhibitor.

Of the 161 patients included in the PFS analysis, 83 (51.6%) patients experienced disease progression, and 78 (48.4%) patients were censored. In this cohort, a total of 13 deaths were recorded. Among these, 12 patients had documented disease progression prior to death, with the time interval from progression to death ranging from 1.0 to 47.5 months. The remaining one patient died on the censoring date without documented progression, and was therefore handled as censored data. No competing events (deaths without prior documented disease progression) were observed in this cohort (**Supplementary Table 6**). With the follow-up period ending on May 1, 2026, the overall median PFS was 28.2 months (95% CI: 23.6–32.8). PR was observed in 77.0% of cases and one patient achieved complete remission. Therapeutic outcomes were stratified by first-line *ALK* inhibitor generation, *EML4-ALK* fusion variant and *TP53* status. First-generation TKI demonstrated significantly inferior efficacy [median PFS: 13.5 months, 95% confidence interval (CI): 5.6–21.4, adjusted *p* = 0.009] compared to second-generation agents (median PFS: 30.8 months, 95% CI: 20.7–40.9), and third-generation TKI (median PFS and 95% CI not reached; **Figure 5a**).

Analysis of *EML4-ALK* fusion variants revealed no significant difference in survival between long-variant and short-variant carriers in the overall cohort (median PFS: 30.6 months vs. 24.3 months, adjusted *p* = 0.60; **Figure 5b**). When stratified by TKI generation, the difference between long- and short-variant carriers was statistically significant only in the second-generation TKI subgroup (adjusted *p* = 0.0189, **Figure 5e**). In a multivariate analysis restricted to the second-generation TKI subgroup, *EML4*-*ALK* variants (long vs. short, HR = 0.370, 95% CI: 0.178–0.770, *p* = 0.008) and *ALK* resistance mutations (HR = 4.824, 95% CI: 1.359–17.116, *p* = 0.015) were independently associated with PFS (**Supplementary Figure 1**). In the first- and third-generation TKI subgroups, no significant differences were observed (adjusted *p* = 1.0, **Figure 5d** and adjusted *p* = 1.0, **Figure 5f**, respectively). The third-generation subgroup was presented descriptively only due to small sample size.

The overall *TP53* effect was not significant after correction for multiple testing (unadjusted *p* = 0.034, adjusted *p* = 0.204, **Figure 5c**). In first-generation TKI subgroup, *TP53*-mutant patients had a significantly shorter PFS than *TP53*-wildtype patients (5.6 months vs. 21.4 months, adjusted *p* = 0.009; **Figure 5g**). In a multivariate analysis restricted to the first-generation TKI subgroup, *TP53*-mutant (HR = 4.152, 95% CI: 1.556–11.080, *p* = 0.004) was independently associated with shorter PFS (**Supplementary Figure 2**). In the second- and third-generation TKI subgroups, no significant differences were observed (adjusted *p* = 0.80, **Figure 5h** and adjusted *p* = 1.0, **Figure 5i**, respectively).

Multivariate Cox regression in overall cohort showed that stage IV disease (vs. stage III, HR = 3.561, 95% CI 1.092-11.614, *p* = 0.035), long *EML4-ALK* variants (vs. short *EML4-ALK* variants, HR = 0.596, 95% CI 0.377-0.942, *p* = 0.027), *TP53* co-mutated (vs. wildtype, HR = 2.269, 95% CI 1.401-3.675, *p* = 0.001), second- and third-generation TKIs (vs. first-generation TKI, HR = 0.276, *p* < 0.001 and HR = 0.172, *p* = 0.001, respectively), and *ALK* resistance mutations (vs. none, HR = 2.541, 95% CI 1.080-5.979, *p* = 0.033) were independent predictors of PFS (**Supplementary Figure 3**).

## Discussion

In this retrospective study, we found marked tumor-type specificity of fusion partners, with *EML4* dominating in LUAD (92.1%), yet it was rarely seen in extrapulmonary tumors (27.3%). 23 novel fusion partners have also been identified. *ALK*-TKIs showed potential activity in patients with rare fusion partners (ORR 76.9%), although this observation is derived from a small, heterogeneous cohort and requires prospective validation. Second- and third-generation TKIs significantly improved PFS over first-generation agents, with generation-dependent prognostic effects of *EML4*-*ALK* variants and TP53 co-mutations. These findings expand the understanding of *ALK* biology and provide real-world evidence to inform treatment decisions.

The detection of *ALK* fusions has traditionally relied on fluorescence *in situ* hybridization (FISH) as the gold standard; however, this method cannot identify specific fusion partners or variants. With the increasing adoption of NGS in clinical molecular profiling, a growing number of novel *ALK* fusion partners have been characterized. As of 2020, 90 *ALK* fusion partners have been reported in NSCLC alone (2). In our study, we identified 15 previously reported *ALK* partners and 23 novel partners, expanding the spectrum of *ALK*-associated alterations. Consistent with prior findings (1), *EML4* was the predominant fusion partner in NSCLC, whereas non-*EML4* partners dominated in extrapulmonary tumors (13).

In our pan-cancer cohort, 13 patients with rare *ALK* fusions showed evidence of clinical activity with *ALK*-TKI therapy, with an ORR of 76.9%. This observation aligns with accumulating case reports demonstrating the efficacy of *ALK* inhibitors across diverse malignancies, including colorectal cancer (14), pancreatic adenocarcinoma (15), neuroendocrine neoplasms (16), renal cell carcinoma (17), prostate cancer (18), inflammatory myofibroblastic tumors (19), mesothelioma (20), and thyroid cancer (21). Furthermore, basket trials have systematically evaluated *ALK* inhibitors in tumors harboring *ALK* aberrations (22), suggesting a potential tissue-agnostic therapeutic activity (23, 24). However, given the limited sample size and the heterogeneous attribution of responses, these findings should be considered hypothesis-generating rather than conclusive. Larger prospective studies are needed to definitively establish the tissue-agnostic potential of *ALK* inhibition in rare fusion-driven tumors.

The differential clinical response to crizotinib based on *EML4*-*ALK* variants was first reported in 2016 (25), with a median PFS significantly longer in patients harboring variant 1 (11.0 months) than those harboring other variants (4.2 months; *p* < 0.05). Subsequent retrospective studies have corroborated these findings and further delineated divergent responses between variant 1 and variant 3, as well as between short- and long-*EML4*-*ALK* variants (26). The phase III ALEX trial (27) provided further evidence, demonstrating that alectinib yielded a median PFS nearly twice as long in patients with variant 1 (34.8 months) compared to those with variant 3 (17.7 months). In contrast, crizotinib showed no significant PFS difference between variant 1 (7.4 months) and variant 3 (9.1 months), a finding consistent with our data. The diminished disparity in crizotinib’s efficacy across variants is likely due to the much shorter PFS observed in patients with variant 1 (26). Strikingly, our analysis revealed that patients with long *EML4*-*ALK* variants exhibited an earlier age of onset than those with short variants, a phenomenon previously reported by Lin et al. (28), but remaining mechanistically unexplained. Further research is warranted to elucidate the biological basis for this association, which could inform therapeutic strategies.

Our study revealed *CDKN2A* and *MDM2* as the most frequent co-occurring genomic alterations in *ALK*-fusion tumors, second only to *TP53* mutations. The *CDKN2A* locus generates two tumor-suppressive proteins through alternative splicing: p16, which directly inhibits CDK4/6 kinase activity, and p14, which stabilizes p53 by competitively binding MDM2 and preventing p53 ubiquitination (29). Notably, *CDKN2A/B* co-alterations correlate with elevated tumor mutational burden, an immunosuppressive tumor microenvironment, and unfavorable prognosis in *ALK*-fusion positive NSCLC (30). This molecular interplay provides a theoretical basis for exploring combination therapy targeting both *ALK* and cell cycle pathways. Preclinical validation comes from studies showing complete regression of *ALK*-aberrant neuroblastoma xenografts with *ALK* and CDK4/6 inhibitor combinations (31), findings now being translated clinically in the NEPENTHE trial (NCT02780128). Collectively, these findings provide a biological rationale for considering *CDKN2A/MDM2* co-alterations as potential biomarkers for future trials of dual-pathway inhibition in *ALK*-driven malignancies, although such approaches remain investigational.

Interestingly, while *TP53* mutations typically predict poorer outcomes with targeted therapies (8–10, 26), we observed no significant PFS difference by *TP53* status in patients receiving second-generation *ALK*-TKI. This apparent discrepancy may be attributable to the limited statistical power due to the relatively small sample size of the *TP53*-mutant subgroup.

While our findings provide important insights into *ALK* fusion-positive cancers, several limitations should be acknowledged. First, as a single-center retrospective study, our cohort is subject to selection bias. The inclusion of biopsy-eligible advanced-stage patients may overestimate PFS and limit generalizability. The co-mutation frequencies may lack generalizability as well. Second, the extended PFS intervals from second- and third-generation *ALK* inhibitors, together with the substantial diversity of subsequent treatments, precluded our ability to report mature overall survival data at the time of analysis. Third, progression assessment relied on routine clinical imaging without independent central review, which may introduce measurement variability and bias in PFS estimation, especially when comparing outcomes across subgroups.

In summary, this study provides a comprehensive real-world characterization of *ALK* fusions across a large Chinese pan-cancer cohort, highlighting the tumor-type specificity of fusion partners, the diversity of rare fusions, and the generation-dependent prognostic impact of *EML4*-*ALK* variants and *TP53* co-mutations. While our findings suggest that *ALK*-TKIs may have activity beyond NSCLC, the small number of extrapulmonary cases precludes firm conclusions about tissue-agnostic efficacy. These findings underscore the value of integrating comprehensive genomic testing into clinical practice to inform personalized therapy for patients with *ALK*-driven malignancies, and highlight the need for larger prospective studies to optimize treatment strategies in this population.

## Data Availability

The datasets presented in this study can be found in online repositories. The names of the repository/repositories and accession number(s) can be found below: https://doi.org/10.57760/sciencedb.010hg.
