# Supplementary material for: Analysis of clinical features and outcomes in a Chinese cohort with ALK fusion across pan-cancer: a retrospective study
Source: Front Oncol. 2026 Sep 3;16:1890263. doi: 10.3389/fonc.2026.1890263 (PMC13581635; doi:10.3389/fonc.2026.1890263)
Supplement: Supplementary file 1 [file SupplementaryFile1.docx]

**Supplemental Information**

*Article title:* *Analysis of clinical features and outcomes in a Chinese cohort with ALK fusion across pan-cancer: A retrospective study*

*Author names:* Meng Zhang^1^, Yi Feng^1^, Xue Du^1^, Changda Qu^1^, Meizhu Meng^1^, Meiying Ye^1^, Min Liang^1^, Ziran Yang^1^, Wenjuan Gong^1^, Xingyu Ma^1^, Jialiang Guo^1^, Wenmei Li^1^, Shuqin Jia^1,*^

*Affiliation and e-mail address of the corresponding author:* Professor Shuqin Jia, Center for Molecular Diagnostics, State Key Laboratory of Holistic Integrative Management of Gastrointestinal Cancers, Beijing Key Laboratory of Carcinogenesis and Translational Research, Peking University Cancer Hospital & Institute, 52 Fucheng Road, Haidian District, Beijing, 100142, China. Tel: +86-010-88196926; Fax: +86-010-88122437; E-mail: jiashuqin2014@163.com

**Supplementary Tables**

Supplementary Table 1 The distribution of cancer types and *ALK* fusion partners across all enrolled patients

| Cancer types | n | *ALK* partners |
| --- | --- | --- |
| NSCLC | 299 |  |
| adenocarcinoma | 292 | *ATP9A, COX7A2L, CRIM1, EML4, EPS15, FNDC3B, KIF5B, KLC1, KMT2E, LRP1B, MAP4K3-DT, MTTP, MYCN, PKDCC, PLEKHH2, PLEKHM2, RRM2, SLC8A1, TFG, VIT* |
| squamous cell carcinoma | 3 | *EML4* |
| adenosquamous carcinoma | 1 | *LRATD1* |
| mucoepidermoid carcinoma | 1 | *HMGN3* |
| large cell lung cancer | 1 | *EML4* |
| carcinoid tumor | 1 | *KCNG3* |
| Extrapulmonary tumors | 22 |  |
| melanoma | 6 | *ADK, DCTN1, DNMT3A, KIF5C, SDHC* |
| sarcoma | 5 | *EML4**, IGFBP5, RANBP2, SORBS1* |
| colorectal cancer | 3 | *ASAP2, EML4, SPTBN1* |
| small cell lung cancer | 1 | *EML4* |
| neuroendocrine tumor | 1 | *EML4* |
| large B-cell lymphoma | 1 | *SEC31A* |
| stromal tumor | 1 | *EML4* |
| hepatocellular carcinoma | 1 | *APOB* |
| cholangiocarcinoma | 1 | *STRN* |
| esophageal gastric junction squamous cell carcinoma | 1 | *ACP1* |
| atypical Spitz tumor | 1 | *MLPH* |

**Abbreviation:** NSCLC, non-small cell lung cancer

Supplementary Table 2 Frequencies of co-occurring genomic alterations in *ALK* fusion‑positive patients, stratified by gene-specific sequencing availability.

| **Gene** | **Patients with available data (n)** | **Patients with alteration (n)** | **Frequency** |
| --- | --- | --- | --- |
| *ALK* | 321 | 16 | 5.0% |
| *EGFR* | 321 | 14 | 4.4% |
| *MET* | 321 | 4 | 1.2% |
| *RET* | 321 | 1 | 0.3% |
| *BRAF* | 320 | 1 | 0.3% |
| *KRAS* | 320 | 3 | 0.9% |
| *PIK3CA* | 320 | 5 | 1.6% |
| *ROS1* | 320 | 1 | 0.3% |
| *ERBB2* | 317 | 3 | 0.9% |
| *PTEN* | 303 | 4 | 1.3% |
| *TP53* | 279 | 101 | 36.2% |
| *AKT1* | 270 | 1 | 0.4% |
| *NRAS* | 270 | 1 | 0.4% |
| *FGFR1* | 264 | 2 | 0.8% |
| *PDGFRA* | 264 | 1 | 0.4% |
| *NTRK1* | 263 | 1 | 0.4% |
| *STK11* | 263 | 0 | 0.0% |
| *CDKN2A* | 262 | 16 | 6.1% |
| *NF1* | 259 | 2 | 0.8% |
| *FGFR3* | 231 | 1 | 0.4% |
| *KIT* | 231 | 2 | 0.9% |
| *APC* | 230 | 4 | 1.7% |
| *RB1* | 230 | 4 | 1.7% |
| *ATM* | 229 | 2 | 0.9% |
| *HRAS* | 229 | 1 | 0.4% |
| *IDH1* | 229 | 2 | 0.9% |
| *IDH2* | 229 | 0 | 0.0% |
| *MLH1* | 229 | 0 | 0.0% |
| *CDH1* | 228 | 0 | 0.0% |
| *CTNNB1* | 227 | 3 | 1.3% |
| *JAK2* | 226 | 0 | 0.0% |
| *GNAS* | 225 | 2 | 0.9% |
| *PTPN11* | 220 | 0 | 0.0% |
| *BRCA1* | 219 | 1 | 0.5% |
| *MTOR* | 219 | 1 | 0.5% |
| *SMARCB1* | 218 | 1 | 0.5% |
| *TSC1* | 218 | 0 | 0.0% |
| *TSC2* | 218 | 1 | 0.5% |
| *CDK4* | 216 | 5 | 2.3% |
| *DDR2* | 216 | 1 | 0.5% |
| *PDGFRB* | 216 | 2 | 0.9% |
| *CDK6* | 213 | 0 | 0.0% |
| *JAK1* | 213 | 0 | 0.0% |
| *MYC* | 213 | 8 | 3.8% |
| *RAF1* | 213 | 0 | 0.0% |
| *VEGFA* | 213 | 1 | 0.5% |
| *NF2* | 212 | 2 | 0.9% |
| *AR* | 209 | 5 | 2.4% |
| *ARID1A* | 209 | 2 | 1.0% |
| *MAP2K1* | 209 | 0 | 0.0% |
| *NTRK3* | 209 | 1 | 0.5% |
| *ERBB3* | 208 | 1 | 0.5% |
| *NFE2L2* | 204 | 3 | 1.5% |
| *BTK* | 203 | 0 | 0.0% |

Frequencies were calculated based on the subset of patients with available sequencing data for each gene due to the use of heterogeneous NGS panels. Genes with fewer than 200 evaluable patients are not shown. The full list is available upon request.

Supplementary Table 3 The relationship between clinical parameters and *ALK* fusion subtypes

|  | *ALK* fusion partner (n, %) | |  |
| --- | --- | --- | --- |
|  | *EML4* | non-*EML4* | *p* value |
| Age (median, IQR) | 52 (44 - 61) | 55 (46.25 - 60) | 0.407 |
| Sex |  |  | 0.932 |
| female | 168 (87.0%) | 25 (13.0%) |  |
| male | 111 (86.7%) | 17 (13.3%) |  |
| Smoke |  |  | 0.977 |
| yes | 67 (87.0%) | 10 (13.0%) |  |
| no | 212 (86.9%) | 32 (13.1%) |  |
| Cancer type |  |  | < 0.001 |
| LUAD | 269 (92.1%) | 23 (7.9%) |  |
| other types of NSCLC | 4 (57.1%) | 3 (42.9%) |  |
| extrapulmonary tumors | 6 (27.3%) | 16 (72.7%) |  |

**Abbreviation: ALK,** **anaplastic lymphoma kinase; EML4,** **echinoderm microtubule-associated protein-like 4; IQR, interquartile range; LUAD, lung adenocarcinoma;** NSCLC, non-small cell lung cancer.

Supplementary Table 4 Clinical characteristics of patients with different variants of *EML4*-*ALK* fusion.

|  | short variants | long variants | *p* value |
| --- | --- | --- | --- |
| Diagnosed Age (median, IQR) | 55 (48 - 63) | 52 (43 - 59) | **0.006** |
| Cancer type |  |  | 1.000 |
| NSCLC | 123 (97.6%) | 150 (98.0%) |  |
| others | 3 (2.4%) | 3 (2.0%) |  |
| Smoking status |  |  | 0.834 |
| non-smoker | 95 (75.4%) | 117 (76.5%) |  |
| smoker | 31 (24.6%) | 36 (23.5%) |  |
| Stage |  |  | 0.312 |
| I-III | 39 (31.0%) | 39 (25.5%) |  |
| IV | 87 (69.0%) | 114 (74.5%) |  |
| Baseline brain metastases |  |  | 0.431 |
| absent | 73 (68.9%) | 91 (64.1%) |  |
| present | 33 (31.1%) | 51 (35.9%) |  |
| NA | 20 | 10 |  |
| Baseline bone metastases |  |  | 0.205 |
| absent | 60 (54.1%) | 91 (61.9%) |  |
| present | 51 (45.9%) | 56 (38.1%) |  |
| NA | 15 | 6 |  |
| Baseline liver metastases |  |  | 0.375 |
| absent | 91 (86.7%) | 118 (82.5%) |  |
| present | 14 (13.3%) | 25 (17.5%) |  |
| NA | 21 | 10 |  |
| Baseline pleural metastases |  |  | 0.709 |
| absent | 66 (59.5%) | 92 (61.7%) |  |
| present | 45 (40.5%) | 57 (38.3%) |  |
| NA | 15 | 4 |  |
| *TP53* mutation |  |  | 0.062 |
| without | 80 (63.5%) | 113 (73.9%) |  |
| with | 46 (36.5%) | 40 (26.1%) |  |
| Resistance mutations in *ALK* |  |  | 0.733 |
| without | 123 (97.6%) | 148 (96.7%) |  |
| with | 3 (2.4%) | 5 (3.3%) |  |

**Abbreviation: EML4,** **echinoderm microtubule-associated protein-like 4; ALK,** **anaplastic lymphoma kinase; SD, standard deviation;** NSCLC, non-small cell lung cancer

Supplementary Table 5 Treatment response of 16 patients bearing rare *ALK* fusion to targeted therapy

| Tumor types | Fusion partners | 1st-line *ALK* TKI | Best response^1^ | Comment |
| --- | --- | --- | --- | --- |
| LUAD | *KIF5B* | crizotinib | SD | The patient received first-line crizotinib, achieving a best response of SD. After 1.6 month, treatment was switched to alectinib, with the best response remaining SD (DOR: 19.4 months). |
| LUAD | *KIF5B* | ensartinib | PR | The patient received first-line ensartinib but switched to alectinib after one month due to AE, achieving a best response of PR (DOR: 27.1 months). |
| LUAD | *KIF5B* | ensartinib | PR | DOR >27.8 months^2^. |
| LUAD | *MYCN* | alectinib | PR | DOR >55.8 months. |
| LUAD | *SLC8A1* | alectinib | PR | DOR 43.4 months. |
| LUAD | *FNDC3B* | ensartinib | NA | Postoperative adjuvant therapy, DOR >39.0 months. |
| LUAD | *COX7A2L* | alectinib | PR | DOR >22.6 months. |
| LUAD | *EPS15* | lorlatinib | SD | DOR >1.1 months. |
| LUAD | *PLEKHM2* | ensartinib | PR | DOR >30.3 months. |
| LUAD | *PKDCC* | alectinib | PR | DOR >25.5 months. |
| ASC | *LRATD1* | alectinib | PR | The patient received first-line alectinib, with a best response of SD (DOR 9.2 months), then switched to lorlatinib due to PD, achieving a best response of PR (DOR >2.8 months). |
| Lung NET | *KCNG3* | alectinib | PR | The patient received first-line treatment with alectinib, with the best response being PR (DOR 18 months), then switched to lorlatinib due to PD, achieving a best response of PR (DOR >6.0 months). |
| LBCL | *SEC31A* | crizotinib | SD | The patient received first-line treatment with crizotinib, with the best response being SD (DOR 3.4 months), then switched to alectinib due to progressive disease, achieving a best response of SD (DOR 48.9 months). |
| CHOL | *STRN* | brigatinib | PR | The patient received first-line treatment with brigatinib, with the best response being PR (DOR 3.4 months), then switched to iruplinalkib for economic reasons, achieving a best response of PR (DOR >18.4 months). |
| IMT | *RANBP2* | crizotinib | NA | Postoperative adjuvant therapy, DOR >9.2 months. |
| MIFS | *IGFBP5* | crizotinib | NA | Postoperative adjuvant therapy, DOR >2.6 months. |

**^1^** Best response refers to the best objective response to *ALK*-TKI therapy overall, rather than specifically to the first-line agent.

^2^ **Values preceded by ">" indicate that the patient remained in remission without disease progression at the last follow-up.**

**Abbreviation:** TKI, tyrosine kinase inhibitor; LUAD, lung adenocarcinoma; SD, stable disease; DOR, duration of response; PR, partial response; AE, adverse effect; ASC, pulmonary adenosquamous carcinoma; Lung NET, lung neuroendocrine tumors; LBCL, large B-cell lymphoma; CHOL, cholangiocarcinoma; IMT, inflammatory myofibroblastic tumor; CR, complete response; MIFS, myxoinflammatory fibroblastic sarcoma.
Supplementary Table 6 Distribution of censoring reasons in the PFS analysis cohort.

| Category | n (%) |
| --- | --- |
| End of study (no event) | 31 (39.7%) |
| Lost to follow‑up | 33 (42.3%) |
| Start of subsequent systemic therapy | 8 (10.3%) |
| Missed radiological assessments | 6 (7.7%) |

**Supplementary Figure 1** Forest plot of multivariate Cox regression analysis for putative risk factors associated with progression-free survival (PFS) in patients treated with second-generation *ALK*-TKIs. Hazard ratios (HRs) with 95% confidence intervals (CIs) are displayed.

**Supplementary Figure 2** Forest plot of multivariate Cox regression analysis for putative risk factors associated with progression-free survival (PFS) in patients treated with first-generation *ALK*-TKIs. Hazard ratios (HRs) with 95% confidence intervals (CIs) are displayed.

**Supplementary Figure 3** Forest plot of multivariate Cox regression analysis for putative risk factors associated with progression-free survival (PFS) in the overall cohort. Hazard ratios (HRs) with 95% confidence intervals (CIs) are displayed.
